# A Survey on Current-Mode Interfaces for Bio Signals and Sensors

**DOI:** 10.3390/s23063194

**Published:** 2023-03-16

**Authors:** Massimo Scarsella, Gianluca Barile, Vincenzo Stornelli, Leila Safari, Giuseppe Ferri

**Affiliations:** Department of Industrial and Information Engineering and Economics, University of L’Aquila, 67100 L’Aquila, Italy

**Keywords:** sensor interface circuits, signal conditioning, biosensors, current-mode approach, Wheatstone bridge, capacitive sensors, resistive sensors, SiPM, VCII, CCII

## Abstract

In this study, a review of second-generation voltage conveyor (VCII) and current conveyor (CCII) circuits for the conditioning of bio signals and sensors is presented. The CCII is the most known current-mode active block, able to overcome some of the limitations of the classical operational amplifier, which provides an output current instead of a voltage. The VCII is nothing more than the dual of the CCII, and for this reason it enjoys almost all the properties of the CCII but also provides an easy-to-read voltage as an output signal. A broad set of solutions for relevant sensors and biosensors employed in biomedical applications is considered. This ranges from the widespread resistive and capacitive electrochemical biosensors now used in glucose and cholesterol meters and in oximetry to more specific sensors such as ISFETs, SiPMs, and ultrasonic sensors, which are finding increasing applications. This paper also discusses the main benefits of this current-mode approach over the classical voltage-mode approach in the realization of readout circuits that can be used as electronic interfaces for different types of biosensors, including higher circuit simplicity, better low-noise and/or high-speed performance, and lower signal distortion and power consumption.

## 1. Introduction

The great success of electronics in many medical and biology-related fields (and not only those) is due to the need for measuring quantities and parameters of biological interest, which is satisfied by the availability of a broad range of existing biosensors. Just as examples, they are used in glucose, cholesterol, pressure, and oxygen level measurements from blood, saliva, or other sources with cheap and commercially available electronics instrumentation, in the detection of the acidity of an aqueous solution, or in quality control processes for the food industry [1,2,3,4,5,6,7,8,9,10,11,12,13,14,15,16]. However, to fully exploit all the great opportunities offered by modern electronic systems, such as the possibility of integrating biosensors and electronics circuits together in an extremely compact system-on-chip, it is necessary to develop conditioning circuits for the detection of bio-signals that enjoy the best possible performances. These conditioning circuits usually represent the interface of the external world with electronics, and are the first and most critical stage of a more complex signal processing system. For example, it might be desirable to make a sensor “smart” by interfacing it with a digital acquisition system through a suitable analog interface. The performance of this electronic interface also affects the operation and overall characteristics of the sensor system. Typical performance requirements are high sensitivity, low-noise operation, wide dynamic range, high-speed performance, low-voltage and/or low power operation, ease of integration on the chip, simplicity of construction, low distortion levels, input–output linearity, etc. As is easy to imagine, the number of possible characteristics can be so great that it is practically impossible to satisfy all of them. For these reasons, the designer usually must make choices considering suitable trade-offs. The classical approach to the realization of simple and reliable electronic interfaces for sensors concerns the use of well-known voltage-mode analog circuits, namely the operational amplifier, which can be employed together with a few additional components to realize readout circuits for the most commonly used and diffused sensors. In this sense, the literature on such voltage mode interfaces is large and detailed [17,18,19,20,21,22,23,24,25,26,27,28,29]. Thanks to this approach, which has eventually become the standard due to the large availability of cheap operational amplifiers, the interfacing of most of the existing sensors with electronics has been possible. As examples, voltage-mode solutions for wide-range resistive sensors are reported in [26,27,28]; in [19,20], techniques for the readout of capacitive ones are described; in [18] an interface circuit is applied to an ISFET; and in [22] an interface for pressure sensors is shown. Even if this approach is successful in many cases, the current-mode techniques have shown that there are situations where the operational amplifier is not the best active block to be used because some of its performances are worse than those offered by the most common current-mode active elements. The main current-mode analog blocks are the second-generation current conveyor (CCII) and its dual, the second-generation voltage conveyor (VCII). These blocks have been successfully applied to the realization of a variety of analog signal processing functions [30,31,32,33,34,35,36,37,38,39,40,41,42,43,44,45,46,47,48,49,50,51,52]. For example, in [31] a current-mode full-wave bridge rectifier has been developed; in [32,40,42] the realization of various filter functions using VCII is shown; and in [33,53] current-mode oscillators are described. It has been shown that the current mode approach has advantages in terms of larger gain-bandwidth products, high-speed operation, low-noise performance, a higher dynamic range, and simplicity of implementation. Moreover, several papers show that the current-mode approach usually requires a lower number of additional components to realize the interface. An example is given in [34], which shows that the minimum number of components to realize an RC sinusoidal oscillator is significantly lower if VCII is used as an active block instead of an operational amplifier. On the other hand, a suitable internal design, at transistor level, is mandatory to reduce the effect of parasitic components, as discussed in [37,54]. For these reasons, the presented work aims to give a review of some of the proposed solutions using these active blocks, the CCII and the VCII, providing a comparison between these circuits and the traditional solutions that use the voltage-mode approach so as to highlight the main benefits of the current-mode approach. However, our discussion is general, and we also make some considerations between the CCII and VCII solutions. The paper is organized as follows: in Section 2 we show the main properties and characteristics of the two main current-mode active blocks used for sensor interfaces; in Section 3 some interfaces that employ these active elements are shown; and in Section 4, comparisons and future prospects are presented. Finally, conclusions are drawn.

## 2. Current Mode Active Blocks for Signal Processing

In this section, we briefly present the two main current-mode active blocks used for the analog processing of signals, namely the second-generation current and voltage conveyors called, respectively, CCII and VCII, which have been successfully applied to the realization of electronic interfaces for bioelectrical sensors and signals [17,18,45,55,55,56,57]. Other current-mode active circuits exist [33,36,40,45,49,55,58,59,60,61,62,63,64,65,66,67,68,69,70], but they will not be considered because they are less known and available and/or their application in biomedical sensor conditioning is not efficient.

### 2.1. Second-Generation Current Conveyor (CCII)

**Figure 1:**
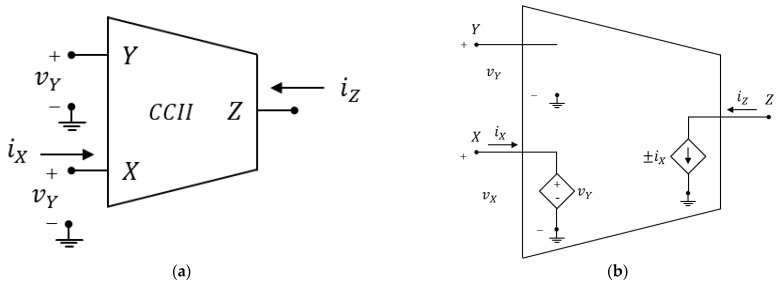
Second generation current conveyor: (**a**) circuit symbol, (**b**) ideal equivalent circuit.

An ideal second-generation current conveyor (CCII) is the 3-port (X, Y, Z) and 4-terminal (including ground) active analog block shown in Figure 1, together with its internal equivalent circuit.

An ideal CCII behaves as a voltage buffer from Y to X and as a current buffer from X to Z, so its ideal current-voltage relationships are:

(1) $$\left[\begin{array}{c} i_{Y}\\ v_{X}\\ i_{Z} \end{array}\right]=\left[\begin{array}{ccc} 0 & 0 & 0\\ \alpha & 0 & 0\\ 0 & \pm \beta & 0 \end{array}\right]\left[\begin{array}{c} v_{Y}\\ i_{X}\\ v_{Z} \end{array}\right]$$

where α and β are unitary gains. Depending on whether it is $i_{Z}=+i_{X}$ or $i_{Z}=-i_{X}$, we distinguish between CCII+ and CCII−, respectively. From the above discussion, Y is a high impedance (ideally infinite) voltage input port; X is a low impedance (ideally zero) current input and voltage output port; and Z is a high impedance (ideally infinite) current output port. However, if a practical implementation of a current conveyor must be used rather than an ideal one, the designer must consider real values for the port impedances and non-unitary gains.

**Figure 2:**
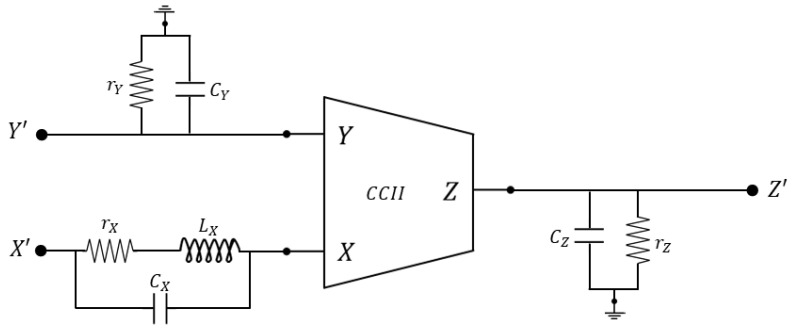
Real second-generation current conveyor.

Figure 2 shows the typical nature of the port impedances and represents a more complete circuit of a real CCII. Equivalent impedances at Y and Z ports are typically of the parallel RC type, while X impedance is of the RLC type, as shown in Figure 2. In most cases, $r_{Y}$ is sufficiently high to be considered negligible. Moreover, other non-ideal effects not discussed here, such as offsets and nonlinearities, have modest entities in most applications, so they can be neglected.

Practical implementations at the transistor level of real CCIIs are reported in [47,71,72]. In [71], a CCII with a rail-to-rail input/output dynamic range is presented, while in [72] the applicability of the concept of adaptive biasing to the CCII is proved. A study of these implementations should clarify the general form of the port impedances in Figure 2. A commercial IC that can be configured as a CCII is the AD844 (fabricated by Analog Devices), which has been used to realize several discrete-component prototypes of CCII circuits. Moreover, we should mention that many generalizations of the CCII as a circuit block have been proposed in the literature [59,60,62,63,64,65,66,67,68,69,70], obtained, for example, when considering differential inputs and outputs or non-unitary or controllable gains, but they have had lower success than the classical CCII illustrated here.

### 2.2. Second-Generation Voltage Conveyor

An ideal second-generation voltage conveyor is nothing more than the dual active circuit block of the CCII [44]. This block is finding increasing applications as an alternative to the well-known voltage-mode circuit and the CCII itself [32,34,35,36,37,38,39,41,43,52,56]. As for the CCII, the information in a VCII is carried both by currents and voltages, but the VCII is equipped with both an input and an output voltage port. This represents one of the main advantages with respect to the CCII, because voltage measurements are generally easier. Other advantages are larger gain-bandwidth products, high-speed operation, low-noise performance, a higher dynamic range, and the simplicity of implementation [44]. Since a VCII is the dual circuit of a CCII, a dual analysis of that illustrated for the CCII can be applied to the VCII.

**Figure 3:**
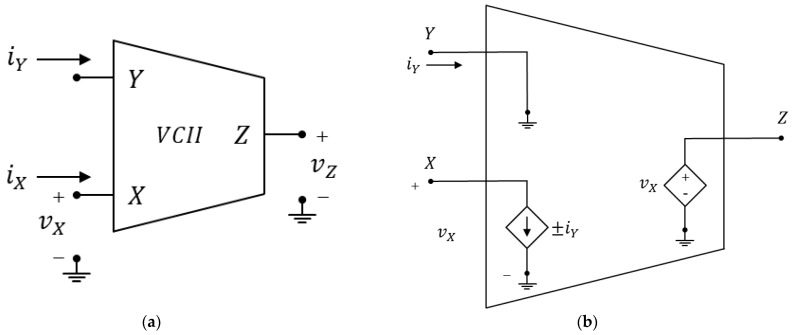
Second generation voltage conveyor: (**a**) circuit symbol, (**b**) equivalent circuit.

**Figure 4:**
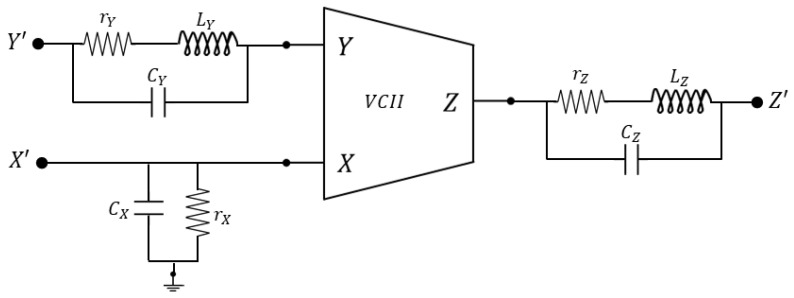
Real second-generation voltage conveyor.

Figure 3 depicts the symbol and the equivalent circuit of the VCII and shows that an ideal second-generation voltage conveyor behaves as a current buffer from Y to X and as a voltage buffer from X to Z. Y is a low impedance (ideally zero) current input port; X is a high impedance (ideally infinite) voltage input and current output port; and Z is a low impedance (ideally zero) voltage output port. The ideal voltage–current relationships of a VCII are the following:

(2) $$\left[\begin{array}{c} i_{X}\\ v_{Y}\\ v_{Z} \end{array}\right]=\left[\begin{array}{ccc} 0 & \pm \beta & 0\\ 0 & 0 & 0\\ \alpha & 0 & 0 \end{array}\right]\left[\begin{array}{c} v_{Y}\\ i_{X}\\ v_{Z} \end{array}\right]$$

where, again, α and β are unitary gains and, depending on whether it is $i_{X}=+i_{Y}$ or $i_{X}=-i_{Y}$, we distinguish between VCII+ and VCII−, respectively. A simple practical implementation at transistor level of a VCII can be found in [73], while more advanced architectures proposed are a low-voltage, high-drive VCII that permits high current drive capability at the X port [74] and rail-to-rail VCIIs, presented in [54,75]. As for the CCII, all these practical implementations of the VCII suffer from non-ideal values of the port impedances and non-unitary gains. Figure 4 shows the typical nature of the various port impedances and represents a more complete circuit of a real VCII. Taking into consideration the duality between CCII and VCII, Y and Z port impedance are of the RLC type, while X impedance is of the RC type, as shown in Figure 4. As for the CCII, the commercial AD844 from analog devices can also be configured as a VCII and used for the implementation of discrete-component circuits.

Finally, we should note that, as for the CCII, some generalizations of the VCII with differential inputs and outputs or non-unitary controllable gain have been proposed [36,39], albeit to a more limited extent with respect to the CCII. This represents a research topic in current-mode processing.

### 2.3. Basic CCII and VCII Configurations

**Figure 5:**
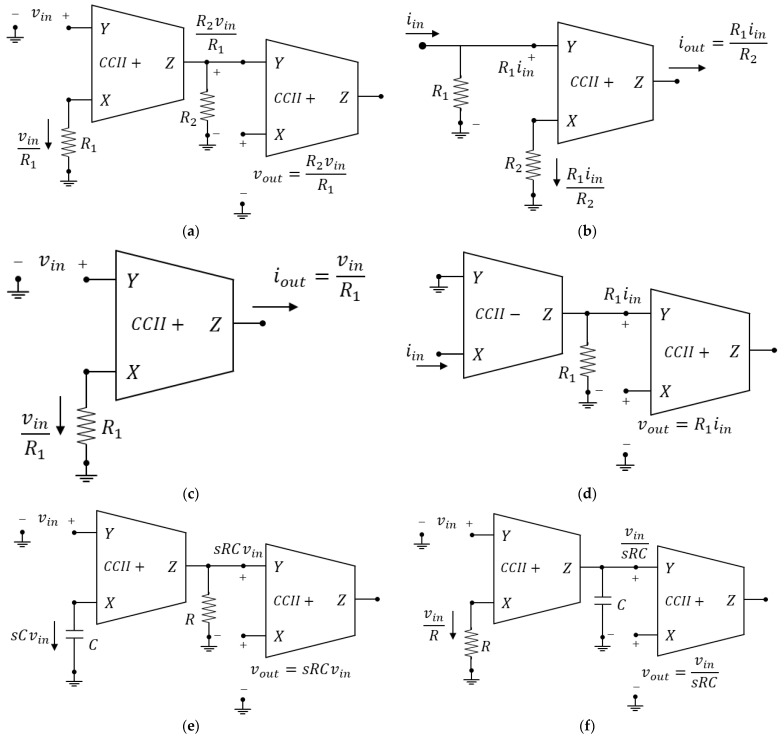

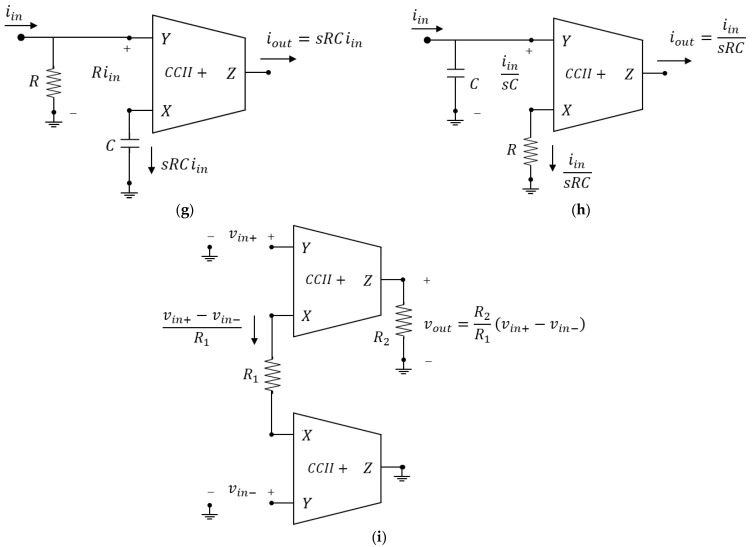
Basic CCII applications: (**a**) voltage amplifier; (**b**) current amplifier; (**c**) V-I converter; (**d**) I-V converter; (**e**) voltage differentiator; (**f**) voltage integrator; (**g**) current differentiator; (**h**) current integrator; (**i**) differential voltage amplifier.

**Figure 6:**
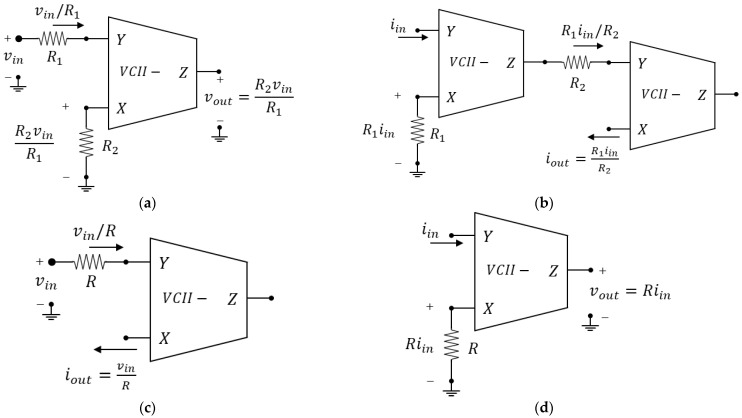

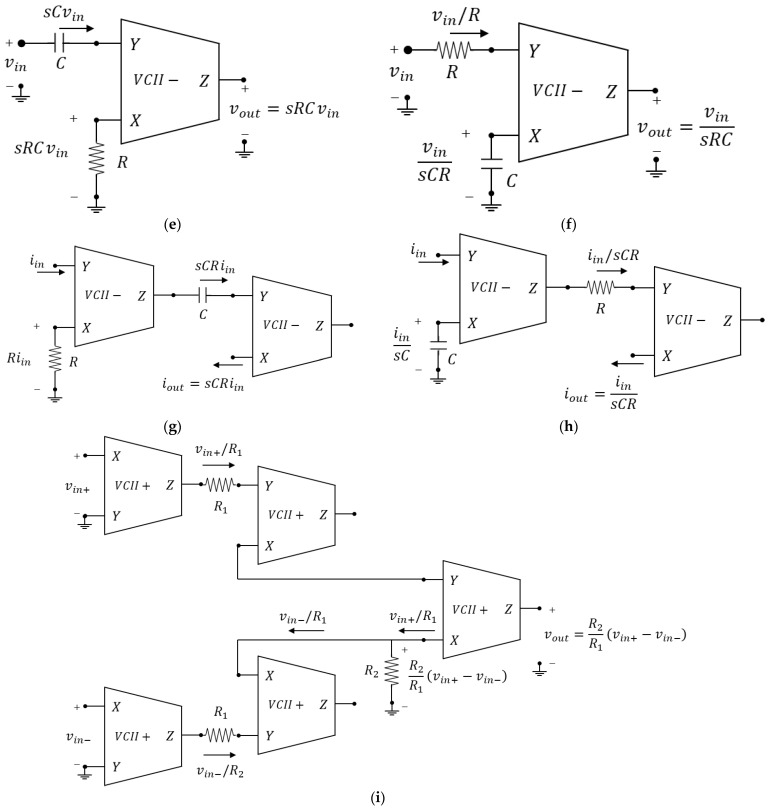
Basic VCII applications: (**a**) voltage amplifier; (**b**) current amplifier; (**c**) V-I converter; (**d**) I-V converter; (**e**) voltage differentiator; (**f**) voltage integrator; (**g**) current differentiator; (**h**) current integrator; (**i**) differential voltage amplifier.

Using VCII or CCII, it is possible to realize common analog signal processing circuits. Depending on the specific application, we find that VCII and CCII are very useful blocks in configurations where classical operational amplifiers are limited, notably in those that require operation on current signals such as current differentiation and integration [44]. Moreover, even in those applications where op-amps provide well-established solutions, CCII and VCII can perform the same tasks but with some important advantages such as higher bandwidth, speed, dynamic range, and lower consumption. The bandwidth improvement is due to the fact that, unlike in voltage mode circuits, where the constant gain-bandwidth product (GBW) represents the main trade-off that limits the obtainable bandwidth, in CCII and VCII this limitation is overcome through the concepts of voltage and current conveying. Since current conveying means unitary gains, the bandwidth is maximized to the GBW value for the current buffer and voltage buffer stages of a CCII or VCII, but at the same time, the availability of a current input or output terminal allows for signal amplification, as shown in the following. Moreover, the current terminal provides even more flexibility than that offered by op-amps, since it makes it easier to perform any current-to-voltage conversion or vice versa. As for the dynamic range improvements, this is because, using current as an information-bearing signal, it is possible to allow high signal swings even with low supply voltages. Finally, as discussed in the following sections, there are many situations where sensors provide a current signal, and the current mode approach is, therefore, more suitable. For these reasons, a brief summary of the basic applications of CCII and VCII is reported in Figure 5 and Figure 6, respectively, which show the most basic circuits commonly used in the conditioning of signals together with standard circuit analysis to explain their operation [44]. In particular, Figure 5a–i show a CCII-based voltage amplifier, current amplifier, transconductance amplifier, transimpedance amplifier, voltage differentiator, voltage integrator, current differentiator, current integrator, and differential voltage amplifier, respectively. Similarly, Figure 6a–i show a VCII-based voltage amplifier, current amplifier, transconductance amplifier, transimpedance amplifier, voltage differentiator, voltage integrator, current differentiator, current integrator, and differential voltage amplifier. A comprehension of such circuits sets the stage for the study of more advanced circuits such as the interfaces presented in the following, and should, hence, prove useful. Other applications not reported here obviously exist and include, for example, oscillators [34], filters [42], and impedance-simulation circuits [36,59].

## 3. Current-Mode Sensor Interfaces for Bioelectrical Signal Conditioning

A transducer is defined as a physical system able to convert a signal from one physical domain to another. Since signal processing is usually most conveniently performed by electronic systems, the output domain is typically the electrical one and in this case, the transducer is defined as a “sensor”. A biosensor is a biologically sensitive device combined with a physical-chemical transducer. A good overview of different biosensors is reported in [76], where biosensor types are classified into electrochemical, optical, thermal, electronic, and gravimetric biosensors, according to the physical–chemical transducer operating principle. As discussed, electrochemical sensors are the most widely investigated and used because they produce signals in terms of voltages, currents, and impedances that are easy to process and measure using electronics systems. A subset of electrochemical sensors is represented by molecular sensors, which can perform molecular recognition in solutions, blood, air, etc. [77]. At the same time, optical biosensors are finding increasing applications because they provide a non-invasive way to measure the same quantities [76,78,79,80,81] and ease in converting optical signals into electrical form. Common examples of electrochemical and optical biosensors are employed in glucose meters, cholesterol meters, and oximetry, which represent commercial, daily-life applications of biosensors. Since the performances of the biosensor are critically affected by the read-out electronic circuit, particular attention must be paid to the choice of the read-out circuit, and the research in current-mode circuits demonstrates the benefits and advantages of this approach to signal conditioning systems [43,52,55,56,72]. For these reasons, in this section we present a review of different current-mode based interfaces suitable for bioelectrical signal conditioning and processing.

### 3.1. Current-Mode Interfaces for Capacitive/Resistive Sensors

**Figure 7:**
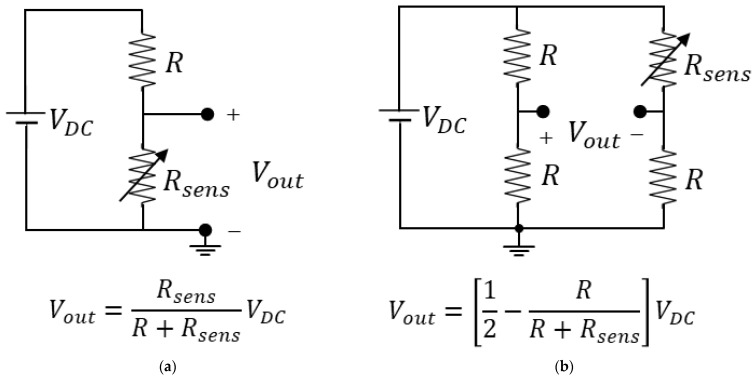
Traditional interfaces for resistive sensors. (**a**) Resistive voltage divider; (**b**) resistive Wheatstone bridge.

**Figure 8:**
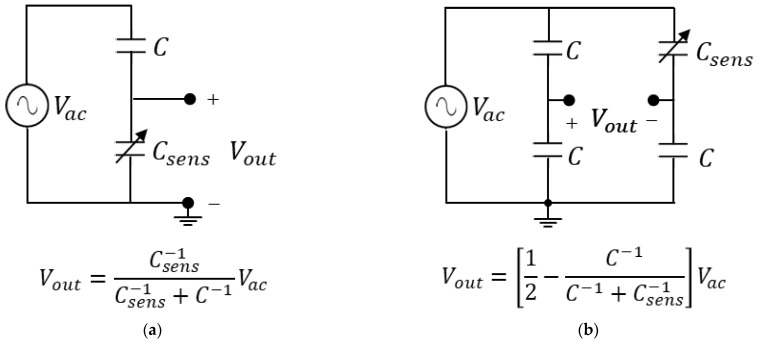
Traditional interfaces for capacitive sensors. (**a**) Capacitive voltage divider; (**b**) capacitive Wheatstone bridge. Here, $V_{ac}$ and $V_{out}$ denote AC amplitude values.

A subset of the class of electrochemical biosensors is represented by capacitive and resistive sensors, which are particular types of electrochemical sensors and constitute a large part of the most common biosensors [75]. In a resistive or capacitive biosensor, the variations of the biological parameter to be measured are converted into impedance variations, in particular resistance or capacitance variations, respectively. An example of a resistive biosensor is represented by blood, whose electrical impedance varies depending on its glucose level [82], while capacitive biosensors have been successfully applied to the detection of proteins, nucleotides, heavy metals, saccharides, small organic molecules, and microbial cells [83]. To maintain circuit simplicity and, hence, the ease of on-chip integration, the traditional voltage approach for the conditioning of such types of sensors consists of inserting them into a simple voltage divider or a Wheatstone bridge that modifies its behavior depending on the measured values, as in Figure 7 and Figure 8. As shown, the divider or bridge may be resistive DC-excited or capacitive AC-excited depending on the sensor’s nature. Although these circuits have proven their potential in implementing cheap and area-saving readout circuits, they suffer from an important limitation that resides in their steady-state (DC or AC) non-linear input–output characteristics. The main implication of this nonlinear behavior is that such circuits cannot be used for large sensor variation since, in each case, circuit output saturates at a fixed supply voltage if sensor impedance ($R_{sens}$ or $1/\omega C_{sens}$) becomes higher than the baseline value (R or 1/ωC, respectively). The sensitivity of an interface circuit is the ratio of the output voltage variation to the sensor resistance or capacitance variation with respect to the baseline value. If the sensor resistance or capacitance varies from its baseline, we may write, respectively:

(3) $$R_{sens}=R+\Delta R=R\left(1+\frac{\Delta R}{R}\right)=R\left(1+\delta_{R}\right)$$

(4) $$C_{sens}=C+\Delta C=C\left(1+\frac{\Delta C}{C}\right)=C\left(1+\delta_{C}\right)$$

where $\delta_{R}$ and $\delta_{C}$ denote ΔR/R and ΔC/C, respectively. The output voltage change in response to such sensor variation for Figure 7a,b and Figure 8a,b, is given by, respectively:

(5) $$V_{out}=V_{DC}\frac{R\left(1+\delta_{R}\right)}{R+R\left(1+\delta_{R}\right)}=V_{DC}\frac{1+\delta_{R}}{2+\delta_{R}}$$

(6) $$V_{out}=V_{DC}\left[\frac{1}{2}-\frac{1}{2+\delta_{R}}\right]=\frac{V_{DC}}{2}\frac{\delta_{R}}{\left(2+\delta_{R}\right)}$$

(7) $$V_{out}=V_{ac}\frac{C^{-1}{\left(1+\delta_{C}\right)}^{-1}}{C^{-1}{\left(1+\delta_{C}\right)}^{-1}+C^{-1}}=V_{ac}\frac{1}{2+\delta_{C}}$$

(8) $$V_{out}=V_{ac}\left[\frac{1}{2}-\frac{C^{-1}}{C^{-1}+C^{-1}{\left(1+\delta_{C}\right)}^{-1}}\right]=V_{ac}\left[\frac{1}{2}-\frac{1+\delta_{C}}{2+\delta_{C}}\right]$$

Hence, the sensitivities are obtained as the following derivatives:

(9) $$\frac{dV_{out}}{d\delta_{R}}=V_{DC}\frac{1}{{\left(2+\delta_{R}\right)}^{2}}\approx \frac{V_{DC}}{4}$$

(10) $$\frac{dV_{out}}{d\delta_{R}}=V_{DC}\frac{1}{{\left(2+\delta_{R}\right)}^{2}}\approx \frac{V_{DC}}{4}$$

(11) $$\frac{dV_{out}}{d\delta_{C}}=-V_{ac}\frac{1}{{\left(2+\delta_{C}\right)}^{2}}\approx -\frac{V_{ac}}{4}$$

(12) $$\frac{dV_{out}}{d\delta_{C}}=-V_{ac}\frac{1}{{\left(2+\delta_{C}\right)}^{2}}\approx -\frac{V_{ac}}{4}$$

The last approximation in (9)–(12) is justified only in the case of a small sensor impedance change with respect to the baseline value, namely if $\delta_{R}<<1$ or $\delta_{C}<<1$. The above equations clearly show that, in all the circuits of Figure 7 and Figure 8, the absolute value of the sensitivity for a small signal is a quarter of the amplitude of the DC or AC stimulus applied. Moreover, as anticipated before, the equations also show that, for high range variation (i.e., $\delta_{R}\gg 1$ or $\delta_{C}\gg 1$), the small-signal sensitivities tend to be very small values as the circuit operations enter into a strongly non-linear compression zone. The sensitivity values may be increased by employing, at the output, a single-input, single-output voltage amplifier for cases Figure 7a and Figure 8a, such as those in Figure 5a or Figure 6a. A similar statement applies to the Wheatstone bridge cases Figure 7b and Figure 8b, using a differential voltage amplifier such as the one in Figure 5i or Figure 6i. The increase in the sensitivity value is given by the gain A of the amplifier.

A simple way to overcome this limitation in the detection of high-variation capacitive or resistive signals is to perform an impedance-to-period conversion, for example by inserting the sensor in a square-wave or generator circuit as an astable multivibrator or in a sine-wave oscillator. Some current-mode architectures have been proposed in this sense. Another interface (exclusive to the current-mode approach) will be discussed in the next subsection, namely the current-mode Wheatstone bridge (CMWB) with intrinsic linearity.

**Figure 9:**
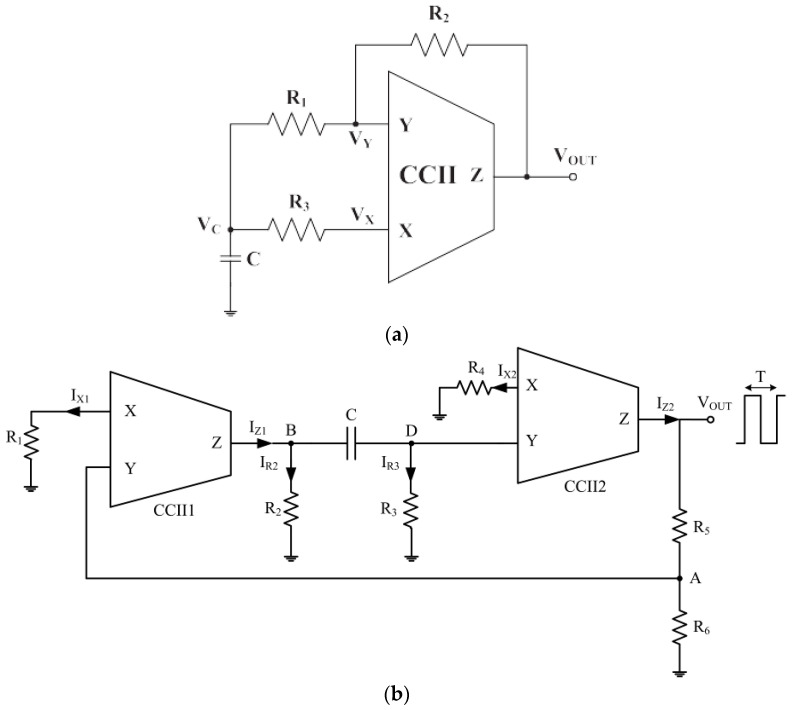

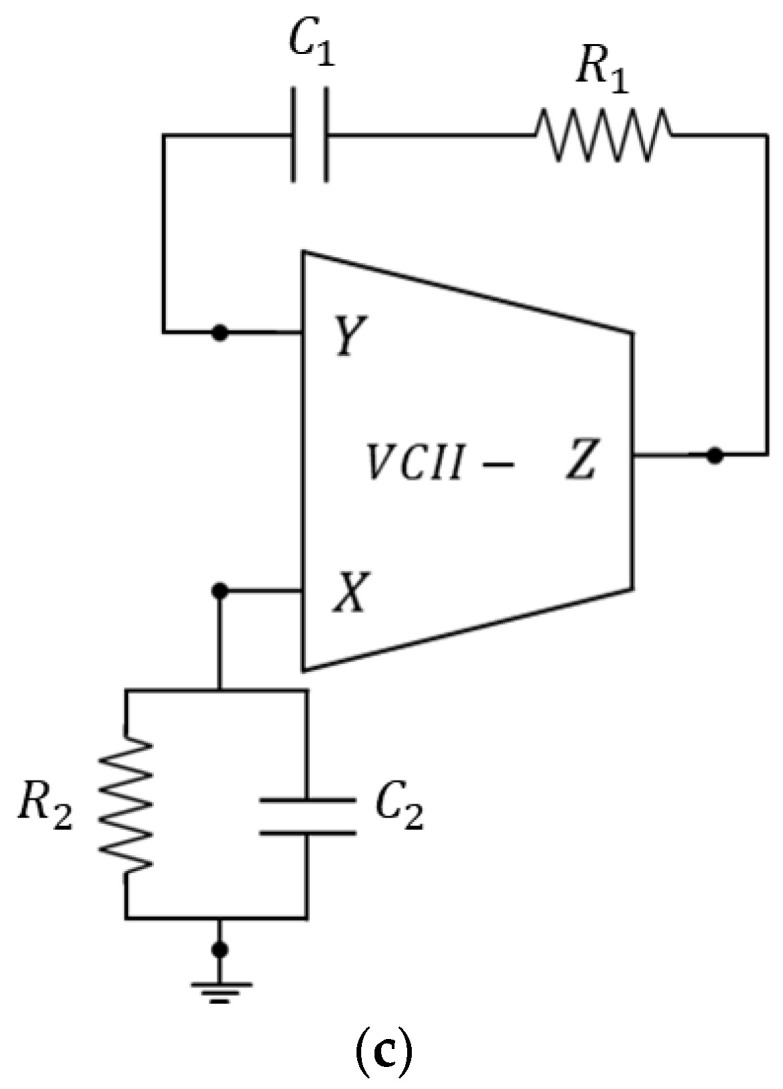
Two different CCII-based proposed interfaces using a square-wave generator for resistive and capacitive sensors: (**a**) the square-wave generator proposed in [53]; (**b**) the square-wave generator proposed in [84]; (**c**) the sine-wave oscillator proposed in [34].

Figure 9a,b show two examples of CCII-based interfaces performing an impedance-to-period conversion for their operation with large sensor variation [53,84]. 

In contrast to previously proposed voltage-mode solutions, these circuits enjoy the advantages of current-mode processing, such as better performance in terms of high-frequency operation. The circuit shown in Figure 9a makes use of just one CCII configured as a Schmitt trigger and capacitor [53]. The period T for an ideal CCII in this case can be approximated by

(13) $$T=2CR_{3}\left[1+\frac{R_{2}}{R_{1}}+\frac{2R_{1}}{R_{1}+R_{3}}\right]$$

It is shown that the circuit approximately maintains this linear C-T relationship and shows relative errors below 10% for capacitance values from 100 pF to 10 uF. The solution proposed in Figure 9b [84] makes use of a current differentiation instead of voltage integration, allowing the estimation of the resistance or capacitance of the sensor while neglecting the effects of both CCII voltage node saturations and internal parasitic components, as follows:

(14) $$T=2C\left(R_{2}+R_{3}\right)\ln\left[\frac{2R_{2}R_{3}R_{6}-R_{1}R_{4}\left(R_{2}+R_{3}\right)}{R_{1}R_{4}\left(R_{2}+R_{3}\right)}\right]$$

According to (13), the period T is linear with respect to C (but not to resistances). For a resistive sensor, typical choices are to replace $R_{2}$ or $R_{3}$ with the sensor because this allows the optimization of the circuit to obtain good linearity. The results show a good agreement between the theoretical and measured periods for a wide range of capacitive values from 10 pF to 10 nF. In both cases Figure 9a,b, the circuits have been experimentally tested using the commercial AD844 from Analog Device, but in [53] simulation results are obtained with a CCII designed at transistor level using the AMS 0.35 um CMOS technology.

Concerning the VCII, Figure 9c shows a possible solution that performs an impedance-to-period conversion [34]. The circuit is the minimal component configuration for a sine-wave oscillator, provided that the sine-wave oscillation condition given by

(15) $$\frac{R_{1}}{R_{2}}+\frac{C_{2}}{C_{1}}=1$$

is satisfied, and the oscillating frequency in such case is

(16) $$f_{0}=\frac{1}{T}=\frac{1}{2\pi \sqrt{R_{1}R_{2}C_{1}C_{2}}}\;.$$

Although the oscillating condition cannot be strictly satisfied if the biosensor replaces just one of the resistances or capacitances in Figure 9c, the circuit still proves useful in this application since it can be shown that, when implemented with a real VCII circuit such as an AD844, it continues to oscillate even when the oscillating condition is released to

(17) $$\frac{R_{1}}{R_{2}}+\frac{C_{2}}{C_{1}}\leq 1$$

although this implies higher total harmonic distortion (THD) values of the output waveform that tend to be more “squared”, and a higher frequency estimation error using Equation (16). The condition (17) is required to ensure that the poles of the circuit transfer function remain on the right side of the complex plane. 

### 3.2. Current-Mode Interfaces for Differential Resistive Sensors 

**Figure 10:**
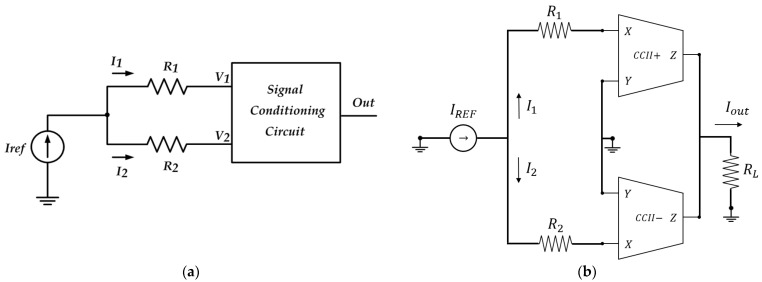

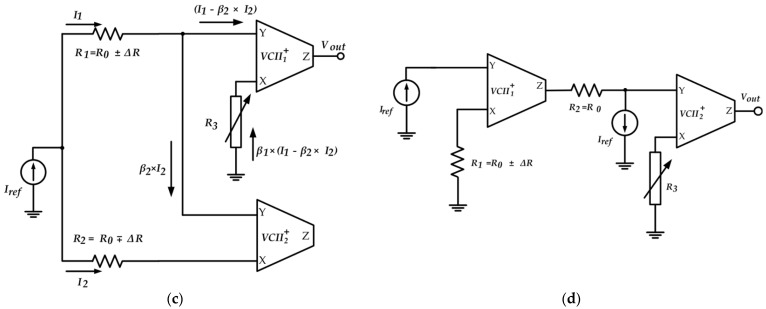
(**a**) The concept of a current-mode Wheatstone bridge; (**b**) CCII-based current-mode Wheatstone bridge; (**c**) VCII-based current-mode bridge for a couple of resistive sensors in differential operation; (**d**) VCII-based current-mode bridge for a single resistive sensor.

To overcome the problems related to oscillator-based interfaces, the recently proposed current-mode Wheatstone bridge in Figure 10a can be used. With respect to the conventional voltage-mode Wheatstone bridge consisting of four resistances, the current-mode Wheatstone bridge [85] has a smaller number of resistors and employs a current instead of a voltage as exciting input, allowing the produced signals to be processed with current-mode active blocks, which enjoy better performances in terms of high-frequency operation. This idea may be applied both to the CCII and VCII cases. For the CCII case [86], the circuit is quite simple and exploits the current inputs of a couple of CCIIs to reduce the complexity of the circuit and take advantage of the high-speed properties of the CCII (Figure 10b). This simple topology may be used to realize a basic interface for a broad range of resistive and differential resistive sensors. As for the VCII, a solution with intrinsic linearity based on the current mode Wheatstone bridge has been developed and applied to resistive sensors [50]. Two topologies are proposed, depending on whether a couple of resistive sensors in differential operation (Figure 10c) or a single resistive sensor (Figure 10d) is used. Both the topologies use VCIIs as active blocks to obtain an easy-to-read voltage output signal that is linearly related to the resistance variations, as given by the following relations (for the cases in Figure 10c,d, respectively).

(18) $$V_{out}\approx \frac{\pm \Delta R}{R_{0}}\alpha_{1}R_{3}I_{ref}$$

(19) $$V_{out}\approx \frac{\pm \Delta R}{R_{0}}\alpha_{2}\beta_{2}R_{3}I_{ref}$$

Equations (18) and (19) also show that the gain can be easily regulated by varying $R_{3}$. The same concept has previously been applied to other current-mode blocks (as the operational floating current conveyors, or OFCC, not discussed here), resulting in circuits that show similar properties in terms of linearity and can be used in situations where a current output instead of a voltage is acceptable. Finally, we should note that even if different circuits have been proposed based on the current-mode Wheatstone bridge, the literature lacks experimental results that show the practical application of such circuits to real sensors.

### 3.3. Current-Mode Interfaces for Differential Capacitive Sensors 

The current-mode Wheatstone bridge has also been applied to differential capacitive sensors, an important subclass of capacitive sensors, for which ad hoc current mode solutions have been designed. A differential capacitive sensor is simply a system of two series capacitances, $C_{1}$ and $C_{2}$, that vary with opposite signs with respect to a baseline value as the measurand varies (as the sensor resistances in Figure 10c do). Biosensors modelled by a differential capacitor exist [87]. The relationship between the measurand x and the two capacitors for these sensors is simply given by:

(20) $$x=\frac{C_{1}-C_{2}}{C_{1}+C_{2}}.$$

**Figure 11:**
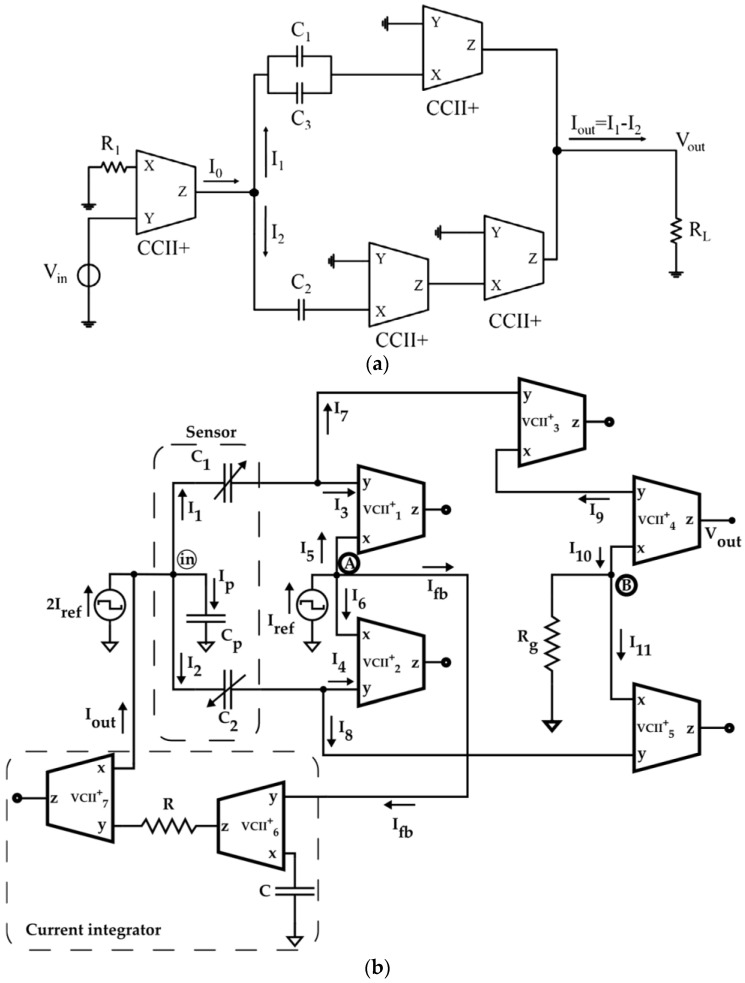
Two different current-mode interfaces for differential capacitive sensors: (**a**) is the CCII-based circuit proposed in [51]; (**b**) is the VCII-based circuit proposed in [52].

Although all the previously shown solutions for capacitive sensors may be successfully applied to differential capacitive sensors, there are some well-known important reasons why these ad hoc solutions are generally better, primarily the mitigation of unwanted common-mode signals and parasitic effects, as well as temperature compensation. Moreover, by properly using a differential capacitive sensor, it is possible to obtain a doubled sensitivity for the interface circuit by exploiting the fact that the two capacitances vary with opposite variation. The use of current-mode blocks for these sensors is justified once again by all the already mentioned benefits that current-mode processing carries with it (e.g., low-power operation, high-frequency performance, increased dynamic range with low distortion, greater implementation simplicity, etc.). Two of the circuits are reviewed here (Figure 11), one based on CCIIs (Figure 11a) [51] and one on VCIIs (Figure 11b) [52].

As discussed in [51], the operation of the circuit in Figure 11a is as follows: $C_{1}$ and $C_{2}$ represent the differential capacitive sensor. The first stage of the interface is just a voltage-to-current converter (added to use the circuit with more common AC voltage inputs), which converts the input voltage $V_{in}$ into a current $I_{0}$. This current ($I_{0}$) is then divided into two parts, $I_{1}$ and $I_{2}$, proportionally to the occurred capacitive variation, and then the currents are subtracted with a couple of CCII to obtain $I_{out}=I_{1}-I_{2}$ and, hence, the voltage $V_{out}$. Performing all the necessary calculations yields the following input–output relation for the peak-to-peak values:

(21) $$V_{out.pp}\left(x\right)=R_{L}^{\prime }\frac{V_{in,pp}}{R_{1}^{\prime }}\left[\frac{C_{0}}{C_{0}+C_{3}}x+\frac{C_{3}}{C_{0}+C_{3}}\right]\;$$

where $R_{L}^{\prime }$ and $R_{1}^{\prime }$ are the resistance values, including the parasitic components of the CCIIs. This equation clearly shows that the simple circuit of Figure 11a has an output peak-to-peak voltage proportional to x, an extremely desirable property that indicates that, in voltage mode processing, more complex circuits are generally required, and shows again the potential of current-mode processing. Equation (21) also clarifies the function of $C_{3}$ as an offset term that is required to distinguish between positive and negative x values (if $C_{3}=0$, the peak-to-peak output voltage has the same values both for negative and positive values).

The analysis now discussed clearly shows two main limitations of the CCII-based circuit, namely that the output is a current signal that requires a load to be converted into a voltage and, more importantly, that the output signal is affected by the internal parasitic capacitance of the sensor $C_{p}$ located between the internal node of the differential capacitive sensor and ground. To overcome these limitations, the VCII solution of Figure 11b [52] can be used since this employs a feedback loop to make the circuit insensitive to stray parameters and does not require a load resistance since the output is on the Z voltage port of a VCII. Its operation is based on exciting the sensor capacitances with a square-wave current signal that is divided into two parts (proportionally to x), plus a third part that flows through $C_{p}$. As discussed in [52], this parasitic capacitance causes a loss of sensitivity in any interface that uses the difference of the two currents through $C_{1}$ and $C_{2}$ in order to evaluate the measurand x. In particular, this can be drastically reduced for small baseline sensors (tens of pF or fewer). The two currents through $C_{1}$, $C_{2}$ are, hence, used to produce an error signal due to this current that is integrated and summed back to the input square-wave current to compensate for the effects of $C_{p}$. At the same time, these currents are also used to obtain an output proportional to their difference, and hence to x. The calculations provide the following simple equation:

(22) $$V_{out}\approx R_{g}I_{reg}x.$$

Results reported show that the full-scale error always remains below 2.5% for a 200 pF baseline linear differential capacitor with |*x*| ranging from 0 to about 90%, while without compensation the error increases with |*x*| until a peak of almost 30%. The corresponding voltage-mode circuit for the conditioning of differential capacitive sensors with parasitic capacitance effect compensation is given in [88]. Comparing the VCII-based solution with the voltage-mode one, we find again that the VCII offers a much simpler solution, so we can say that the results of the interfaces in Figure 11 prove the high potential of CCIIs and VCIIs in implementing low-cost, highly accurate, and fully integrable read-out circuitry for differential capacitive sensors.

Until now, we have discussed electronic interfaces for different types of resistive and capacitive sensors. Starting from the next section, we consider some examples of more specific electronic circuits for the conditioning of biosensors that do not belong to these classes. 

### 3.4. A Current-Mode Interface for ISFET Sensors 

**Figure 12:**
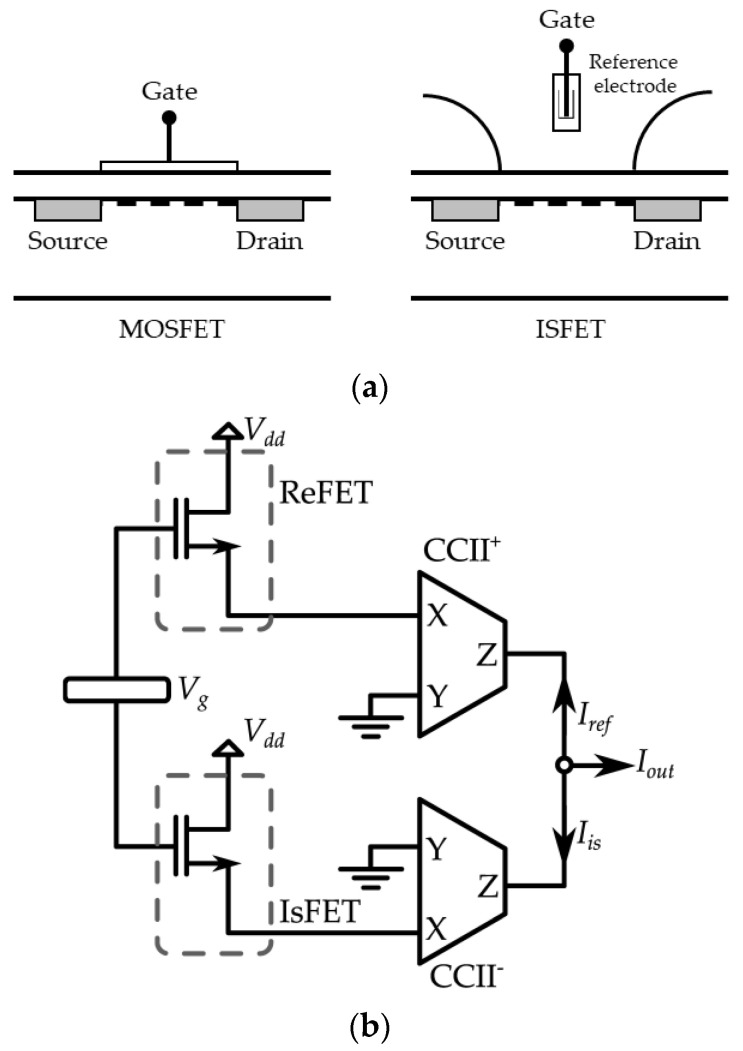
(**a**) Comparison of ISFET and MOSFET; (**b**) standard differential current-mode approach.

The concept of current-mode Wheatstone Bridge has also been applied to the conditioning of ion-sensitive field-effect transistor (ISFET) sensors. As the name suggests, ISFET sensors are FET-based sensors where the current that flows through the FET varies with the ion concentration in a solution, and are frequently used in biological applications as a pH sensor [89]. The physical structure and operation are easy to understand if we compare the ISFET sensor with the well-known MOSFET. As shown in Figure 12a, an ISFET is nothing more than a MOSFET with a remote gate separated from the chip, for which the threshold voltage is linearly related to the pH. Just like a MOSFET, the ISFET can operate in linear or saturation region depending on the DC voltages at its terminals. By biasing the ISFET in the linear region, the current will be linearly related to the threshold voltage and, hence, to the pH, an extremely desirable property. The proposed current-mode interface for ISFET sensors is shown in Figure 12b and employs just two CCIIs, the ISFET sensor, and another reference transistor REFET that has the same electrical performance as the ISFET but is not sensitive to the ions in the solution. Since this reference transistor is biased in the linear region to the same working point as the ISFET and has the same temperature dependence as the ISFET, the circuit realizes temperature compensation and the output current $I_{OUT}=I_{ISFET}-I_{REFET}$ does not vary as the temperature varies. A comparative analysis of this circuit with other voltage-mode readout circuits [18] for ISFET highlights that the first one is more suitable to apply to micro-sensors, since it requires a lower voltage supply, consumes less power, and has a higher response speed. Moreover, having a current output can be an advantage in those cases in which the output signal has to be converted into digital form, since current is more suitable for A/D conversion.

### 3.5. A Current-Mode Interface for Silicon Photomultipliers

**Figure 13:**
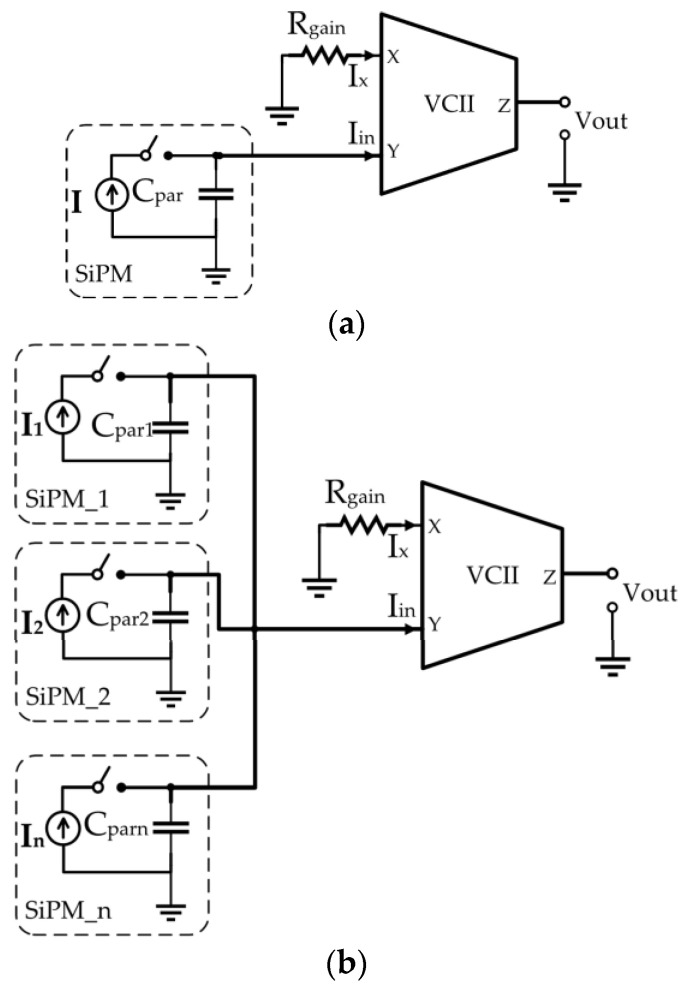
VCII-based interface circuits for (**a**) a single SiPM and (**b**) an array of SiPMs.

Silicon photomultipliers (SiPMs) are becoming an interesting alternative to other electronic light detectors and classical photomultiplier tubes (PMTs) since they are able to combine an extremely high sensitivity with the advantages of possible integration with other sensors and circuits on a system-on-chip, and they are finding increasing applications as biosensors [90,91]. The design of an appropriate interface circuit for these sensors is not an easy matter since it is required to deal with their relatively high output capacitance, which can reach values up to thousands of pF for an array of SiPMs. This means that the readout circuit must have the lowest possible input impedance to minimize the effects of such parasitic capacitance. Moreover, an appropriate circuit should fulfill, at the same time, other important requirements such as high linearity, fast response time, and low-noise performance, and it is in these requirements that current-mode processing can play an important role. In fact, all the conventional voltage-mode readout circuits [92,93], based mostly on common-gate/common-base amplifiers and operational amplifiers, suffer from limitations in terms of circuit complexity and/or high-frequency and low-noise performances that make these structures unattractive. A recently published work [48] shows that the current-mode technique can be successfully applied to the design of high-speed and low-noise interface circuits. The proposed circuit is shown in Figure 13a for a single photomultiplier and in Figure 13b for an array of SiPMs. The output of the circuit in both cases is given by:

(23) $$\frac{V_{out}}{I_{in}}=\alpha \beta R_{gain}$$

where α and β are the voltage gain and current gain of the VCII. The extreme simplicity of these circuits, which use just one resistor and a VCII to obtain a transimpedance amplifier, allows the minimization of the cost and the noise of the circuit at the same time. Most importantly, this current-mode solution exhibits, for the first time, the interesting property that the achieved gain is independent of bandwidth because, differently from operational amplifiers, the VCII has a constant bandwidth. If the noise minimization is also carried out at transistor level using an appropriate internal structure for the VCII optimum noise performance can be obtained, and the study carried out in [73] presents the main guidelines that must be considered for an optimum design. Finally, we must note that even though it is reasonable to assume that similar performances can be obtained using CCIIs, a corresponding study on the implementation of a CCII-based interface for SiPMs is missing.

### 3.6. Current-Mode Interfaces for Ultrasonic Sensors 

**Figure 14:**
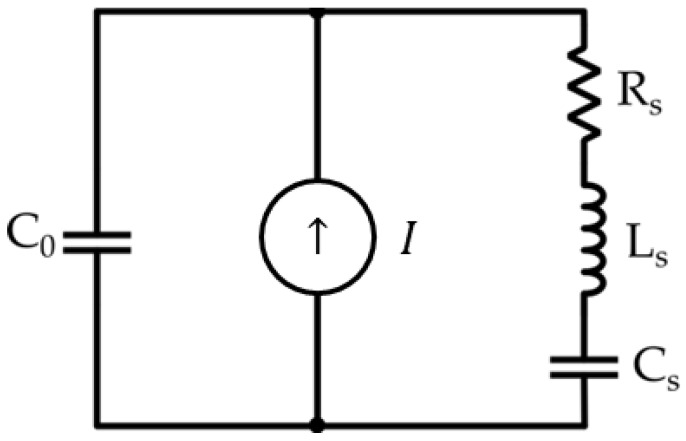
Equivalent circuit of a piezoelectric transducer in reception. The current I is the derivative of the charge that develops in response to an applied force.

Another important class of sensors is that which generates and receives sound waves in the ultrasonic band, commonly known as ultrasonic sensors. Although not a biosensor in a strict sense, ultrasonic sensors are finding increasing application in the biomedical field. For example, a noteworthy application is their use in echography for human body imaging [94]. The most popular approach for the task of ultrasonic signal reception and generation is to use a piezoelectric crystal such as PZT or PVDF [95,96]. When a piezoelectric crystal is excited with an AC voltage in the ultrasonic band, it slightly compresses and expands following the AC stimulus signal and transmits such mechanical oscillations to the surrounding medium (whether it is air or something else). Vice versa, a piezoelectric crystal can also be used for signal reception of ultrasonic mechanical waves, as discussed here. When used in reception, the piezoelectrical crystal is an active sensor typically represented by a generator and a filter network composed of resistances and reactive elements, as in Figure 14. Here, $C_{0}$ represents the static capacitance of the transducer, while the series RLC branch models high-frequency resonance. However, for use as a sensor, the signal frequencies are typically such that this latter branch can be neglected. The current source is simply the derivative of the charge q that develops at the two ends of the crystal, being thus proportional to applied force:

(24) $$I=\frac{dq}{dt}\;.$$

An alternative representation with a voltage source is clearly possible, but it is preferable to represent the sensor as a current generator in this context because its impedance is typically somewhat higher than the impedance of the Y input port of a VCII, a fact that allows directly interfacing the spiral shaped PVDF sensor to the VCII. This has been exploited in [46], where a circuit similar to that in Figure 13a for a SiPM has been applied to a spiral-shaped omnidirectional PVDF sensor specifically designed to receive ultrasounds in air in a wide frequency range (20–80 kHz) [97]. The designed circuit displays a transimpedance gain of 86 dBΩ and an overall power consumption of about 6 mA. The reported PVDF sensor sensitivity is in the range between −107 and −101 dB, with a beam directivity of 360° on both the vertical and horizontal planes. As well as in the SiPM case, the same advantage, that the achieved gain is independent of bandwidth, remains obviously valid.

**Figure 15:**
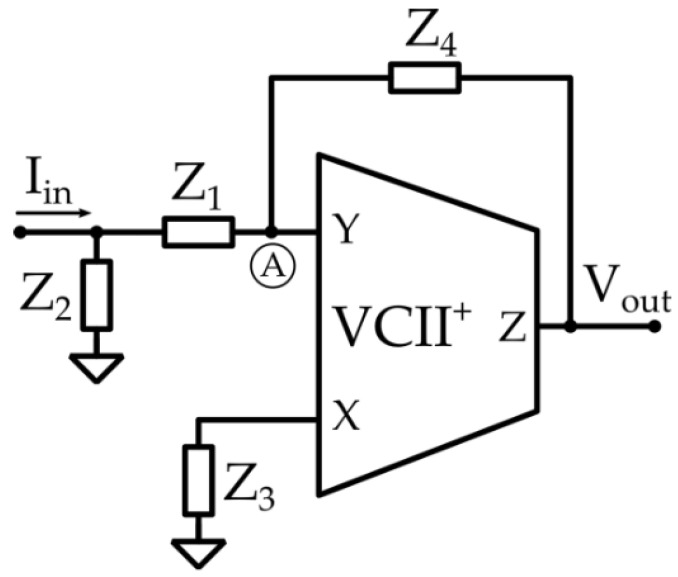
VCII-based second-order low-pass and bandpass filter.

In [46], a circuit to perform a noise filtering function in addition to transimpedance amplification was developed to obtain a purified version of the received signal, depicted in Figure 15. This goal was achieved using $Z_{1}=1/sC_{1}$, $Z_{2}=R_{2}$, $Z_{3}=1/sC_{3}$, $Z_{4}=R_{4}$ to obtain a second-order band-pass filter, with a transfer function given by

(25) $$\frac{V_{out}}{I_{in}}=\frac{sC_{1}R_{2}R_{4}}{1+s\left(C_{1}R_{2}+C_{3}R_{4}\right)+s^{2}C_{1}C_{3}R_{2}R_{4}}$$

or, alternatively, using $Z_{1}=R_{1}$, $Z_{2}=1/sC_{2}$, $Z_{3}=R_{3}$, $Z_{4}=1/sC_{4}$ to obtain a second-order low-pass filter with transfer function

(26) $$\frac{V_{out}}{I_{in}}=\frac{R_{3}}{1+s\left(C_{2}R_{1}+C_{4}R_{3}\right)+s^{2}C_{2}C_{4}R_{1}R_{3}}$$

where the sensor impedance has been considered negligible in calculations. 

## 4. Comparison and Future Prospects

In this section, we present a not-exhaustive comparison of the performance of current-mode interfaces with respect to conventional ones. Table 1 shows a comparison between some current-mode oscillator-based interfaces and conventional voltage-mode ones. A quick inspection of this table should clarify the main benefit of the current-mode approach for this category of readout circuits. Using the current-mode approach, it is generally possible to obtain comparable or sometimes even better performances in terms of dynamic range and sensitivity with a simpler circuit. This aspect becomes even more important if the readout circuit is to be integrated on a system-on-chip, since it is advantageous in terms of area occupation of the chip. Unfortunately, as discussed in previous sections, although a VCII may be used to realize such types of readout circuits without any particular problem, the literature on this kind of VCII application is lacking, and an extensive study on this has to be performed. As shown, most of the solution in Table 1 uses OAs, CCIIs, or OTAs, although many other active blocks may be obviously used.

Concerning the differential capacitive sensor case, Table 2 compares the current-mode solution (Figure 11) with others in the literature for this sensor type. The VCII- and CCII-based circuits provide good comparable performance in terms of sensitivity and measurement range, but with simpler topologies like those considered in Section 3.3.

Related to SiPM sensors, Table 3 compares other solutions with the VCII SiPM interfaces of Figure 13. Due to the distinctive property of constant bandwidth of the VCII, very high transimpedance gain and low power consumption are obtained. Moreover, the VCII-based circuit can easily perform the current summing operation (due to the low-impedance current input port) that is very useful in SiPM read-out circuits. Unfortunately, a similar comparison for the CCII case is not possible because no such CCII applications have been reported in the literature until now.

## 5. Conclusions

In this paper, some examples of traditional and current-mode read-out circuits for various types of sensors and bioelectrical signals are analyzed and compared. The comparison shows that the processing of signals in the current domain makes VCII and CCII more flexible with respect to other active building blocks. These circuits may be applied to a broad range of sensors, from the widespread biosensors now used in glucose/cholesterol meters and in oximetry to more specific sensors such as ISFETs, SiPMs, and ultrasonic sensors. The study results indicate that current-mode read-out circuits have several benefits. Among these, we have intrinsic linearity, very simple read-out circuits for sensors configured in the CMWB, better accuracy with parasitic-insensitive operation for differential capacitive sensors, and very simple read-out circuitry for SiPM and PVDF sensors with extended bandwidth and low power operation.

## Figures and Tables

**Table 1:** Comparison between some current-mode oscillator-based read-out circuits and conventional voltage-mode ones.

Ref.	Dynamic Range	Employed Components	Sensitivity	Output Waveform
[98]	C (floating): 4.7 pF–2.2 uF R (floating): 820 kΩ–9.1 MΩ or 500 kΩ–5.95 MΩ	2 OA 6 R 1 C	C (floating): 21 us/pF R (floating): 50–90 ms/MΩ or 170–900 ms/MΩ	square
[53] ^1^	C (grounded): 22 pF–5.5 uF R (floating): 47–470 kΩ	1 CCII 3 R 1 C	C (grounded): 178 ns/pF R (floating): 30 us/kΩ	square
[99]	C (floating): 1–47 pF R (floating): 100 kΩ–100 GΩ	4 OA 1 XOR gate 3 R 2 C	Not available	square
[100]	R (grounded): 0.7–7 kΩ	2 OA 4 R 1 C	R (grounded): 330 us/kΩ	square
[101]	C (floating): 0.8–1.2 pF	4 OTA 1 OA 1 AND 3 C	C (floating): 15–47 us/pF	square
[102]	C (floating): 1–22 pF R (floating): 10 kΩ–1 GΩ	4 OTA 1 AND gate 7 R 3 C	C (floating): 330 us/kΩ	square/ triangular
[103]	C (floating): 0–33 pF R (floating): 470 kΩ–100 GΩ	4 OTA 1 XOR gate 3 R 2 C	C (floating): Not available R (floating): 320 us/MΩ	square
[104] ^1^	R (floating): 10 kΩ	1 CCII 3 R 1 C	R (floating): 222 Hz/kΩ	square
[105] ^1^	R (floating): 0.2–1 kΩ C (grounded): 10 nF–20 uF	2 CCII 3 R 2 C	R (floating): 3.1 kHz/kΩ C (grounded): 0.02 Hz/pF	square/ triangular
[106] ^1^	R (floating): 2–100 kΩ C (floating): 500 pF–10 uF	2 CCII 3 R 1 C	R (floating): 250 us/kΩ C (floating): 4 ns/pF	square/ triangular

^1^ Current-mode circuit.

**Table 2:** Comparison between current-mode read-out circuits for differential capacitive sensors and conventional ones.

Ref.	Baseline Capacitance	Approach	Sensitivity	Variation Range
[51] ^1^	400 pF	C to V conversion	Not available	±30%
[52] ^1^	10–200 pF	Mixed	412/21 mV/pF	±100%
[107]	140 pF–14 nF	C to V conversion	71 mV/pF	±100%
[108]	500 pF	C to V conversion	5 mV/pF	±50%
[109]	400 pF	C to digital conversion	4 counts/pF	±50%
[30]	1 pF	C to I conversion	50 nA/fF	±100%
[110]	20 pF	C to V conversion	833 mV/pF	±60%
[111]	400 pF	C to V conversion	nonlinear	±100%
[112]	250 pF	C to freq. conversion	0.8 kHz/pF	±60%
[113]	1 pF	C to I conversion	Not available	±75%

^1^ Current-mode circuit.

**Table 3:** Comparison between current-mode SiPM read-out circuits and conventional ones.

Ref.	Tech	Supply	Power	T-I Gain	Bandwidth	Noise
[48] ^1^	CMOS 130 nm	1.2 V	0.34 uW	100 dB	10 MHz	27 m$V_{rms}$ (output)
[114]	CMOS 350 nm	3.3 V	0.68 uW	100 dB	50 MHz	1300 e-(ENC)
[115]	CMOS 350 nm	3.3 V	0.68 uW	500	150 MHz	2 u$V_{rms}$ (input)
[116]	SiGe 130 nm	−3.2 V	82 uW	56 dB	45 GHz	30.6 $\frac{pA}{\sqrt{Hz}}$
[117]	CMOS 180 nm	1.8 V	743 uW (16 channels)	Not available	Not available	56.48 dB (SNDR)
[118]	CMOS 350 nm	3.3 V	380 mW (36 channels)	Not available	Not available	Not available
[119]	CMOS 180 nm	Not available	Not available	10 dB	5 MHz	Not available

^1^ Current-mode circuit.

## Data Availability

Not applicable.
